# Localized assembly for long reads enables genome-wide analysis of repetitive regions at single-base resolution in human genomes

**DOI:** 10.1186/s40246-023-00467-7

**Published:** 2023-03-09

**Authors:** Ko Ikemoto, Hinano Fujimoto, Akihiro Fujimoto

**Affiliations:** Department of Human Genetics, Graduate School of Medicine, The University of Tokyo, Hongo 7-3-1, Bunkyo, Tokyo, Japan

**Keywords:** Long reads, Nanopore, ONT, Localized assembly, Insertions, Structural variation, Variant

## Abstract

**Background:**

Long-read sequencing technologies have the potential to overcome the limitations of short reads and provide a comprehensive picture of the human genome. However, the characterization of repetitive sequences by reconstructing genomic structures at high resolution solely from long reads remains difficult. Here, we developed a localized assembly method (LoMA) that constructs highly accurate consensus sequences (CSs) from long reads.

**Methods:**

We developed LoMA by combining minimap2, MAFFT, and our algorithm, which classifies diploid haplotypes based on structural variants and CSs. Using this tool, we analyzed two human samples (NA18943 and NA19240) sequenced with the Oxford Nanopore sequencer. We defined target regions in each genome based on mapping patterns and then constructed a high-quality catalog of the human insertion solely from the long-read data.

**Results:**

The assessment of LoMA showed a high accuracy of CSs (error rate < 0.3%) compared with raw data (error rate > 8%) and superiority to a previous study. The genome-wide analysis of NA18943 and NA19240 identified 5516 and 6542 insertions (≥ 100 bp), respectively. Most insertions (~ 80%) were derived from tandem repeats and transposable elements. We also detected processed pseudogenes, insertions in transposable elements, and long insertions (> 10 kbp). Finally, our analysis suggested that short tandem duplications are associated with gene expression and transposons.

**Conclusions:**

Our analysis showed that LoMA constructs high-quality sequences from long reads with substantial errors. This study revealed the true structures of the insertions with high accuracy and inferred the mechanisms for the insertions, thus contributing to future human genome studies. LoMA is available at our GitHub page: https://github.com/kolikem/loma.

**Supplementary Information:**

The online version contains supplementary material available at 10.1186/s40246-023-00467-7.

## Background

Long-read sequencing technologies, such as Oxford Nanopore Technologies (ONT) and Pacific Biosciences (PacBio), have the potential to overcome the limitations of next-generation sequencing technologies and provide a comprehensive picture of the human genome [1]. Current technological shortcomings, such as high sequencing error rates, are steadily being overcome, and many recent studies using long reads have interrogated previously inaccessible regions [2–4]. Further, long-read technologies have enabled the identification of pathogenic structural variants (SVs) such as large deletions in *EYS* in retinitis pigmentosa [5] and the expansion of a triplet repeat in *NOTCH2NLC* in neuronal intranuclear inclusion disease [2]. Moreover, there is a growing demand for clinical applications of long reads studies to solve common and Mendelian disorders [1]. Studies using long reads may thus contribute to new therapeutics and more accurate diagnosis of human disease.

However, many problematic regions are still harbored in the human genome. Widely distributed repeats, such as tandem and interspersed repeats, can cause issues in the data processing [6]. Importantly, expansions of tandem repeats (TRs) cause various disorders [2, 7, 8], and transposable elements (TEs), including *Alu* element and LINE, have been associated with a range of disorders [9, 10].

Recently, the first complete sequence of a human genome was finished by the Telomere-to-Telomere (T2T) Consortium [11], which added 238 Mbp of non-syntenic sequences to the GRCh38 assembly. This study also showed that most additional bases were derived from repetitive sequences, such as centromeric satellites and segmental duplications, suggesting that previous research based on de novo assembly may have dismissed a large portion of repetitive sequences. Although the T2T-CHM13 assembly is one of the greatest achievements of long reads, it was attained by sequencing the cell line from a complete hydatidiform mole (uniformly homozygous) and by combining data from multiple platforms including HiFi, ONT, Illumina, and other state-of-the-art techniques. Such large-scale, comprehensive approaches cannot be applied to practical clinical studies in most cases due to cost and human resources.

Current SV calling is essentially based on two types of methodologies: a de novo assembly-based approach and a mapping-based approach [12]. One of the advantages of the former lies in the detection of large variants [12, 13]. However, this method is hampered by haplotype representations and assembly errors caused by repetitive sequences [14]. On the other hand, a mapping-based approach with long reads is advantageous when coverage of the available data is low or the samples contain low-frequency SVs [12]. The variant discovery of this approach usually begins with the mapping of reads to a reference genome and detects SVs based on specific mapping patterns. However, misalignments due to sequencing errors sometimes cause false positives and negatives, and tangled, nested SVs prevent accurate SV calling. Considering these advantages and disadvantages of the current approaches, we aimed to establish a hybrid approach to analyze SVs at single-base resolution.

Here we developed a localized assembly tool, LoMA (Localized Merging and Assembly), which generates accurate consensus sequences (CSs) based on the mapping results of long reads. LoMA captures haplotype structures based on SVs and produces haplotype-resolved CSs, which helps identify heterozygous variants. To our best knowledge, only one tool, lamassemble, has been developed to fulfill a similar purpose [15]; however, this software cannot classify heterozygous regions, which can hinder the resolution of human diploid genomes. In response, we implemented haplotype classification of a target region to improve the accuracy of SV detection.

We applied LoMA to genome-wide SV detection in two samples using the high-coverage whole-genome sequencing (WGS) data from ONT. Generally, true genomic structures of insertions are difficult to resolve. Thus, we aimed to reveal true inserted sequences at single-base resolution. Our analysis showed that LoMA constructs high-quality sequences from single-platform long-read data with substantial errors and revealed the true structures of tandem and interspersed repeats in the human genome.

## Methods

### Development of LoMA

**Fig. 1:**
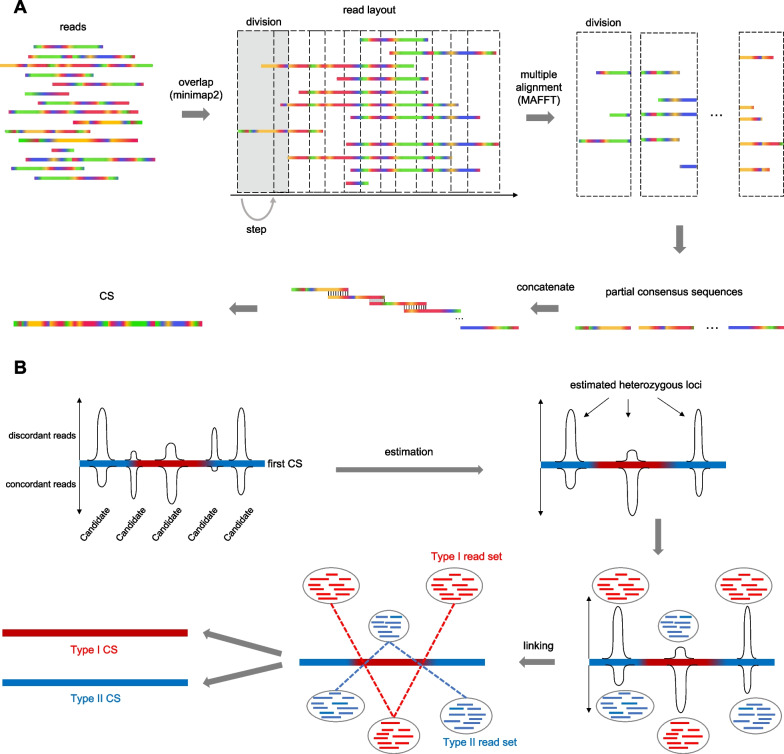
Schematic overview of LoMA's algorithm. **A**. The main process to compose a consensus sequence (CS) is shown. Reads are laid out based on all-to-all alignment using minimap2 [16]. The layout is then subdivided into small divisions for the successive procedure to determine partial CSs based on multiple alignments using MAFFT for each division [17]. The entire CS is obtained by concatenating these small divisions. **B**. The read classification process is shown. Reads constituting a CS are aligned to the CS using minimap2 [16]. LoMA predicts heterozygous loci in the region based on the number of reads supporting a SV, and the reads derived from each estimated haplotype are gathered (Type I and II read sets). Both sets return to the main process (**A**) after this step

Raw reads yielded by ONT are accompanied by substantial sequencing errors. LoMA is a tool to assemble long reads mapped to a region of interest and generates a highly accurate CS that spans the target region (Fig. 1). LoMA detects heterozygous SVs in a target region and outputs haplotype-resolved sequences. In the first step, LoMA constructs a CS spanning the region. This process is initiated by finding overlaps of raw reads using all-to-all pairwise alignment using minimap2 (-x ava-ont) [16] and then generates a layout of overlapped reads (read layout) (Fig. 1A). Based on the all-to-all pairwise alignment, the read layout is estimated. First, pairs with a dangling read or short overlapping lengths are excluded to remove possible alignment errors (Additional file 2: Fig. S1). Second, the longest read is selected, and pairwise alignments are used to determine the positional relationship between the longest read and other reads. The read layout is divided into overlapped, evenly-spaced divisions of constant length, and read sequences included in each division undergo a multiple alignment using MAFFT [17]. Based on the multiple alignment, each consensus nucleotide is determined at every position, and a partial CS is generated for each division. Finally, partial CSs of neighboring divisions are aligned using MAFFT and concatenated into a single CS spanning the entire target region.

The generated single CS can be a mosaic sequence (pseudo-haplotype) mixing paternal and maternal haplotypes, resulting in the dismissal of one allele of the heterozygous variants. To overcome this problem, we adopted the read-separation step (Fig. 1B). In this step, all input reads are aligned to the first CS recurrently by using minimap2 [16]. Then, bins (700 bp) with mismatch clusters are detected based on two conditions: the number of discordant reads to the first CS being > 7 and the number of discordant reads being within the mean ± 3σ of a binomial distribution with *p* = 0.5 (see Additional file 2). Two haplotypes that have multiple heterozygous SVs at different loci are separated using reads that span more than one SV (Fig. 1B). By tracking back all heterozygous bins in a target region, all reads are coherently classified into two individual read sets (Type I and II) in most cases (Fig. 1B). Two CSs are generated from Type I and II. Two read sets will not be produced if a target region does not contain any SV, and the region will be regarded as homozygous.

### DNA sequencing and data processing

Two samples, NA18943 and NA19240, from the International HapMap Project, were analyzed [18]. NA19240 is a Yoruban male sequenced with PromethION (ONT) by De Coster et al. [19]. The WGS data of this sample were downloaded in fastq files from the European Nucleotide Archive (accession number: PRJEB26791) [20]. NA18943 is a Japanese male and is the first Japanese sample sequenced by short reads [21]. In this study, its genomic DNA was extracted from a B cell line and sequenced for 26 flowcells using MinION (ONT).

Basecalling was performed for the WGS data of NA18943 using Guppy (ver.4.4.1) [22]:

--flowcell FLO-MIN106 --kit SQK-LSK109 --device auto

The sequencing data of both NA18943 and NA19240 were then aligned to the GRCh38 assembly using minimap2 (ver.2.0-r290-dirty) [16]:

-a -g2000 -A1 -B2 -O2,32 -E1,0 -z200

### Accuracy assessment and simulation of LoMA

To estimate the accuracy of LoMA, we compared CSs assembled using the ONT data of NA18943 with GRCh38. We randomly selected 108 positions from the human genome while excluding centromeres and gaps (Additional file 1: Table S1). We collected all reads mapped within 20 kbp of each position from the data of NA18943 and constructed CSs using LoMA. We aligned the generated CSs to GRCh38 using minimap2 [16] and calculated the error rates from the edit distance. We also aligned all raw reads to GRCh38 and calculated error rates for the raw reads again using the edit distance. For a comparison, we assembled matched regions using lamassemble [15]:

-P 8 -a -v -p 2e-3 -m 2*(number of reads) -z 1000 promethion.mat

The error rate of lamassemble was calculated as above.

We also evaluated LoMA using simulated data. We randomly selected one hundred regions from GRCh38 (Additional file 1: Table S2). Simulated reads were generated using NanoSim with the error profile of NA12878 (total error rate, 10.8%) provided by the developers [23]. Various data sets were generated for each region: coverage 10, 20, 30, 40, and 50 (with a fixed size of 20 kbp), targeted size 20 kbp, 40 kbp, 60 kbp, 80 kbp, and 100 kbp (with a fixed mean coverage of 30×). The error rate, CPU time, and peak memory (RSS) were measured. A computer with M1 chip (Apple) was used to measure the performance. The error rate (edit distance) was calculated as described above.

### Detecting unclear regions in genomes

**Fig. 2:**
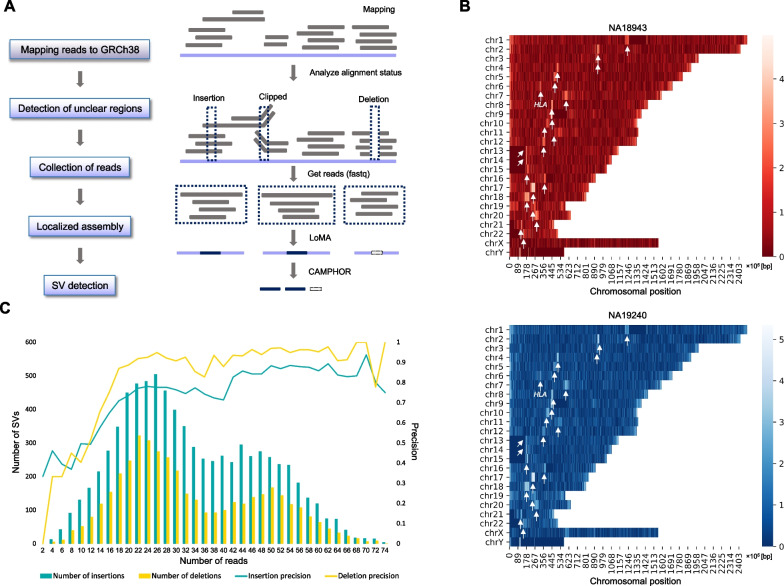
Whole-genome LoMA analysis. **A**. The workflow of the whole-genome analysis. Unclear regions were first defined based on the alignment status (indels and clips) of ONT reads. Reads mapped to the regions were separately collected. For each region, LoMA attempted a localized assembly to obtain CSs. CAMPHOR collectively detected structural variants from the CSs for NA18943 and NA19240. **B**. The relative density of unclear regions of NA18943 and NA19240 are shown in red- and blue-colored heatmaps, respectively. The light-colored regions are dense with unclear regions. The white arrows indicate the autosomal centromeres except chromosome 6. The arrows on chromosome 6 represent the *HLA* region. **C**. The precision of indels to the standard SV set was assessed for NA19240. The left vertical axis (bar graphs) shows the number of indels found in each bin (the number of constitutive reads). The right (line graphs) shows the precision of indels in each bin. Both graphs were binned per 2 reads

To detect SVs from the two samples, we applied LoMA to the WGS data. We first searched for target regions (unclear regions) by scanning all chromosomes from telomere to telomere. We split each autosome and sex chromosome binned per 500 bp, step size 250 bp, and defined an “unclear” region as follows: (1) average coverage between 10 and 200, (2) total number of reads containing indels (≥ 100 bp) or hard- or soft-clipped sequences (≥ 500 bp) > 10, and (3) the proportion of reads containing indels (≥ 100 bp) or hard- or soft-clipped sequences (≥ 500 bp) > 0.2 (Fig. 2A). Then, multiple bins within 10 kbp were merged into one bin. We defined each merged bin as an unclear region in this study. After defining the unclear regions for both NA18943 and NA19240, we collected reads mapped within 10 kbp from both ends of each unclear region using SAMtools [24].

### Localized assembly and SV detection

To obtain CSs of the unclear regions defined above, we analyzed fastq files by LoMA and constructed CSs with the following parameters:

-b3000 -s2000 -h10 -d3 -r0.5 -m1000

We then called indels (≥ 100 bp) found in the CSs using CAMPHOR [3].

### Experimental validation and benchmark of SV detection

To evaluate the accuracy of inserted sequences obtained from CSs, we performed Sanger sequencing. We randomly selected 121 homozygous insertions from NA18943. PCR primers were designed in flanking regions, and the PCR-direct sequence was conducted. We compared the sequences by Sanger sequencing with the matched sequences of CSs generated by LoMA. Since the length of homopolymers was difficult to determine by Sanger sequencing, the stretches of poly(A) were removed from the comparison.

An accurate SV callset of NA19240 was released (the standard SV set) in a previous study leveraging multi-platform data [25]. We compared the indels of our SV calling result (the LoMA SV set) with those of the standard SV set and assessed the precision. We considered variants from the LoMA SV set as concordant (true positive) with the standard SV set if both were the same variant class and the distance between two breakpoints of the SVs was < 500 bp.

### Decomposing insertions

Insertion events are known to be caused by various mechanisms and have various consequences [26]. To characterize and investigate the origins of the detected insertions, we decomposed them into TRs, TEs, tandem duplications (TDs), satellite sequences, dispersed duplications, processed pseudogenes, alternative sequences, “deletions” in GRCh38, and nuclear mitochondrial DNA sequences (NUMTs).

We first applied Tandem Repeats Finder (TRF) [27] to all inserted sequences and defined TRs as having (1) element lengths < 50 bp and (2) covering more than 50% of an inserted sequence. After filtering TRs, we identified TEs using RepeatMasker [28] if (1) an inserted sequence covered a TE > 50%, (2) the inserted sequence was covered by the TE > 50% (reciprocal overlap), and (3) the total substitutions and indels were < 50% (matching condition).

Previous studies have reported that TDs are understudied but widespread [26, 29]. After detecting TRs and TEs, we manually reviewed the remaining insertions and found that they contained TDs derived from non-repetitive regions in the reference. We considered these insertions as TDs. To identify this class of insertions, we aligned all insertions except TRs to GRCh38 using BLAT [30]. We then collected insertions mapped to original breakpoints within 5 bp with > 90% in BLAT identity and defined them as TDs. In this process, missing TRs with long repeat elements were found. Therefore, they were added to the TR callset if (1) an inserted sequence aligned within 500 bp from the insertion breakpoint and (2) the ratio of the total number of matching bases to the insertion length was > 0.5.

To understand the remaining insertions, we manually checked their features by aligning them to the reference using BLAT [30]. We identified insertions that were aligned from end to end to different chromosomal regions with high identity (> 90%). We defined these insertions as dispersed duplications. Next, we detected insertions aligned to a series of exons and untranslated regions (UTRs) of coding genes with high identity (> 90%) and classified them as processed pseudogenes. We also found other insertions aligned to the alternative sequences (e.g., “alt” or “fix” sequences) on BLAT with high identity (> 90%). We classified them as alternative sequences. Some of the insertions left at this point were thought to have arisen by deletion events in GRCh38 because they were securely aligned to the chimpanzee reference genome (panTro6), although they were classified as insertions when compared with GRCh38 [3]. We aligned the remaining insertions to the panTro6 assembly and categorized the insertions that lifted over panTro6 with high accuracy (> 90%) within 100 bp of the inserted position on GRCh38 as "deletions” in GRCh38. After this, the remaining insertions were manually reviewed, and features of the genomic regions (segmental duplications or self-chain) were examined.

### Features of tandem repeats

TRs are well known to have a high mutation rate [31]. We investigated the expansion rates of TRs by comparing them with the reference (GRCh38) as follows:

$$\frac{{{\text{Length}}\;{\text{of}}\;{\text{inserted}}\;{\text{TR}} + {\text{Reference}}\;{\text{TR}}\;{\text{length}}}}{{{\text{Reference}}\;{\text{TR}}\;{\text{length}}}}$$

To investigate the composition of expanded elements, we next analyzed repeat element lengths and unit sequences for short tandem repeats (STRs, 2–6 bp). We indiscriminately treated a group of STRs that were the same in lexicographical order without discriminating strands (see Additional file 2). We next analyzed TR expansions in coding genes using the definition of the RefSeq gene database [32]. We also analyzed expansions in TEs and surrounding regions. We referred to the repeat-annotated GRCh38 sequence by RepeatMasker [28] and counted the number of TR expansions detected inside, upstream (< 100 bp) and downstream (< 100 bp) of TEs. Binomial tests were performed to examine the bias in the frequency of TR expansions upstream and downstream of SINEs.

### Features of target site duplications in Alu element

Several TE families, including L1, *Alu* and SVA (SINE-VNTR-*Alu*) elements, remain active in the human genome [33]. In the integration process of an *Alu* element, a duplicated sequence is copied and inserted at the flanking site, which is called the target site duplication (TSD) [34]. This is the hallmark of retrotransposition. By surveying *Alu* insertions accompanied by TSDs, we analyzed TSD characteristics. We made a non-redundant *Alu* insertion set by merging the results of the NA18943 and NA19240 (merging condition: pairwise distance < 500 bp). We then used the MEME Suite [35] to find motifs at the first and second nicking sites. We also analyzed the length distribution of the TSDs of *Alu* elements.

### Features of tandem duplications

We next explored an underrepresented class of insertions, TDs. TDs are the duplication of a single copy sequence. However, the mechanism of its generation remains unclear. We analyzed the expression levels of genes with and without TDs using GTEx data [36] and examined differences using the Wilcoxon rank-sum test. We also performed binomial tests to analyze the association of TDs with TEs based on the repeat annotation of GRCh38 [28] and with coding genes based on the RefSeq gene data [32].

## Results

### Sequencing data

We sequenced the genomic DNA of NA18943 from a B cell line using a single platform, MinION (ONT). After combining yields from 26 flowcells, the sequencing data totaled 231.6 Gbp (77 × coverage) (Additional file 1: Table S3). The downloaded data of NA19240 was 255.8 Gbp (79 × median coverage) [19].

### Reduction in error rate and accuracy validation of LoMA

**Fig. 3:**
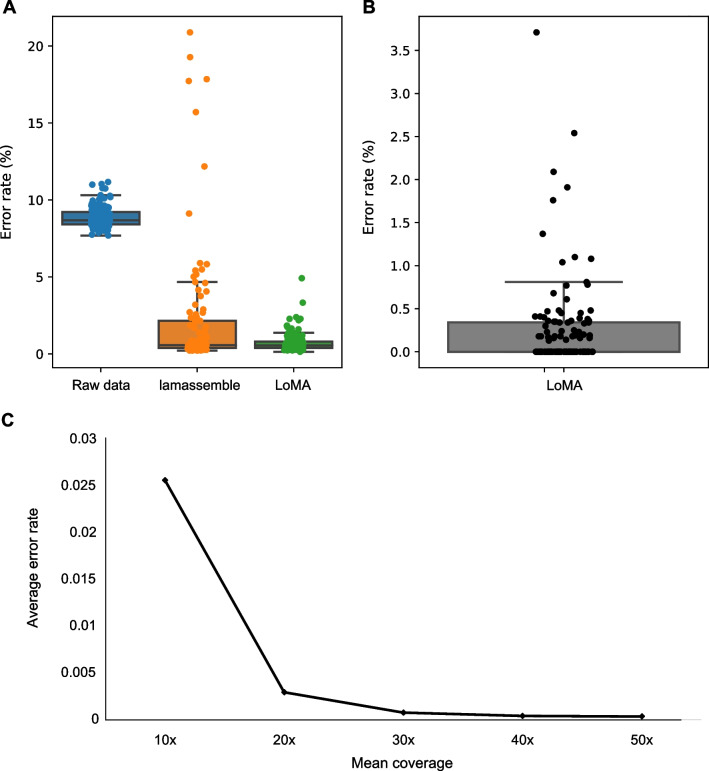
Accuracy assessment and simulated error rate of LoMA**. A.** A comparison with raw reads (blue) and sequences by lamassemble (orange). Consensus sequences (CSs) by LoMA (green) showed a sharp drop in the error rate compared with raw reads and lamassemble. The standard deviations were 0.67, 4.09 and 0.72 for LoMA, lamassemble and raw reads, respectively, demonstrating that the dispersion of LoMA is smaller than of lamassemble and that the instability of sequences in raw data is reduced by LoMA. **B.** A comparison of CSs constructed by LoMA with sequences by Sanger sequencing. The sequences from 121 insertions of NA18943 showed high accuracy (at least 99.7%). Individually, 66 out of 121 (54.5%) regions constructed by LoMA were error-free compared with poly(A) compressed sequences. **C**. The average error rate of simulated data (n = 100). Simulated reads were generated for one hundred randomly selected genomic regions and analyzed by LoMA. The average error rates were 2.6% (10×), 0.29% (20×), 0.076% (30×), 0.041% (40×), and 0.034% (50×) for each mean coverages from 10 to 50

To evaluate the accuracy of LoMA, we performed extensive validations. First, we randomly selected 108 regions and constructed CSs using LoMA (Additional file 1: Table S1). Thirteen of the 108 regions were classified as heterozygous, and two CSs were generated for each heterozygous region. In total, 121 CSs were obtained. We aligned them to GRCh38 and assessed the error rate. The total alignment length was approximately 7.3 Mbp (Additional file 1: Table S4). The estimated error rate was 8.7% (SD = 0.72) for raw reads and 0.76% (SD = 0.67) for CSs by LoMA (Fig. 3A, Additional file 1: Table S1). Similarly, we attempted to construct CSs of matched regions using lamassemble for a comparison but failed to assemble three of the 108 regions, leaving 105 obtained sequences (Additional file 1: Table S1). The alignment with GRCh38 estimated the mean error rate of the lamassemble sequences to be 2.3% (SD = 4.1) (Fig. 3A, Additional file 1: Table S1).

We next performed an experimental validation for the CSs generated by LoMA. We found 14 SV candidates from 13 heterozygous regions (Additional file 1: Tables S1, S5). The primer design and PCR amplification were successful for eight heterozygous candidates, and the product sizes of all candidates were concordant with the expectation of LoMA (Additional file 1: Table S5, Additional file 2: Fig. S2).

Further, we performed Sanger sequencing for 121 homozygous insertions randomly selected from NA18943 (Additional file 1: Table S6). The total compressed length amounted to 66,126 bp, and 65,932 bp were matched to CSs by LoMA (sequence identity 99.71%) (Fig. 3B, Additional file 1: Table S6). Sixty-six CSs from the 121 insertions (54%) were perfectly matched (identity 100%) to the corresponding Sanger sequences (Additional file 1: Table S6).

To evaluate the computation performance of LoMA, we performed a simulation using the ONT read simulator, NanoSim [23]. The results of the reconstruction of randomly selected regions (n = 100) showed an error rate of 2.6% for 10×, 0.29% for 20×, 0.076% for 30×, 0.041% for 40×, and 0.034% for 50× (Fig. 3C, Additional file 1: Table S2, Additional file 2: Fig. S3). The computation time and peak RSS of LoMA were also measured. The computation time was generally linear for coverage up to 50× and target size up to 100 kbp, with a processing time of a few minutes (Additional file 1: Table S7, Additional file 2: Figs S4, S5). The peak RSS was approximately 1 GB for coverage up to 50× and target size up to 100 kbp, and the memory increase was more suppressive than linear (Additional file 1: Table S8, Additional file 2: Figs S4, S5). These results suggest that LoMA had sufficient performance for the sequence analysis.

### Genome-wide analysis of SV polymorphisms using LoMA

To detect SVs using LoMA, we identified unclear regions based on the number of reads with clipped sequences or indels (Fig. 2A and Methods). In NA18943, we detected 27,562 bins as candidates, and they were combined into 16,544 unclear regions. In NA19240, 56,158 candidate bins were detected and combined into 18,928 unclear regions. The larger number of unclear regions in NA19240 reflected the high genetic diversity of African populations [37]. Both NA18943 and NA19240 showed a similar pattern of the landscape of unclear regions: they were localized in the pericentromeric and *HLA* region in both samples (Fig. 2B).

We next constructed the sequences from the unclear regions. After the LoMA analysis, we obtained the CSs of 13,822 and 15,220 regions for NA18943 and NA19240, respectively, including 3065 and 7542 heterozygous regions (see Additional file 2).

### Comparison with a standard SV set and threshold for SV detection

The benchmark with a standard SV set of NA19240 indicated that the precision decreased under the coverage of 20×, although it became stable after more than 20 reads (Fig. 2C). The mean precisions of insertions and deletions with 20 or more reads were 0.82 and 0.93, respectively. For less than 20 reads, the mean precision was 0.52 for each. However, the percentage of SVs with less than 20 reads in the entire SV set was 21.9% for insertions and deletions, respectively; thus, excluding these SVs did not strongly affect the conclusions of this study. Accordingly, we focused on SVs in regions with 20 or more reads to define a more conservative callset of indels. After this filtration, we finally obtained 5516 insertions and 2687 deletions in NA18943, and 6542 insertions and 3475 deletions in NA19240 (the LoMA SV set).

### Overview of insertions and deletions

**Fig. 4:**
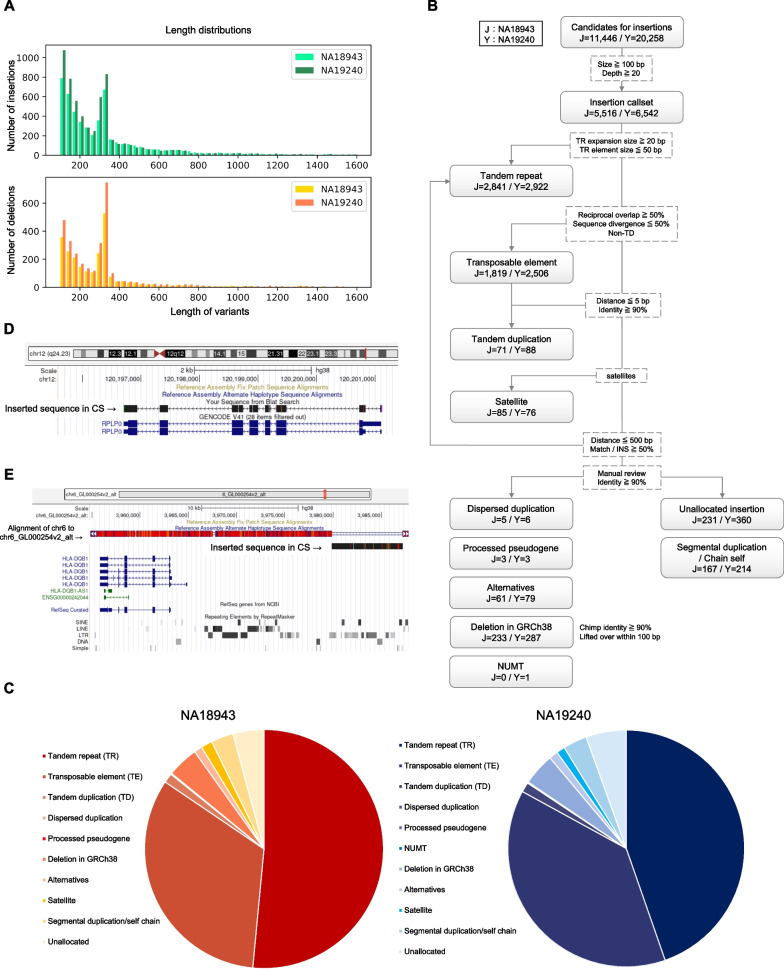
Overview of the variant detection.** A**. The length distributions of insertions (top) and deletions (bottom). The maximum length was capped at 1600 bp. **B**. The flowchart shows the breakdown of the insertions. Dotted-line boxes represent the conditions of classification, and solid-line boxes are each class of insertions. **C**. The pie charts show the proportion of each class of insertions of NA18943 (left) and NA19240 (right). Insertions were decomposed into multiple classes: TRs (tandem repeats), TEs (transposable elements), TDs (tandem duplications), dispersed duplications, processed pseudogenes, NUMT, “deletions” in GRCh38, alternative sequences, satellites, and others. **D**. A processed pseudogene (*RPLP0*) detected in NA19240 is shown (in black). The picture was extracted from Genome Browser. The BLAT identity was 99.9% compared with the reference gene, and the alignment pattern of 5′ UTR showed a closer resemblance with the splicing variant shown at the bottom. **E**. An insertion detected in NA19240 matching the alternative sequence is shown (in black). The picture was extracted from Genome Browser. The breakpoint was 15 kbp upstream of *HLA-DQB1*. LoMA constructed part of the alternative sequence accurately (99.8% in BLAT identity)

The length distributions of the indels were consistent with a previous study (Fig. 4A) [3], suggesting that the genome-wide analysis using LoMA provided a reliable result. Sequence analysis at single-base resolution is effective for understanding the biological mechanism of insertions. To understand the characteristics of the detected insertions, we first classified them (Fig. 4B, C and Methods). The numbers of TRs were 2841 (51.5%) and 2922 (44.7%) in NA18943 and NA19240, respectively, TEs totaled 1819 (33.0%) and 2506 (38.3%), and TDs amounted to 71 (1.3%) and 88 (1.3%). Approximately 1.5% (NA18943) and 1.2% (NA19240) of the insertions were attributed to variants in satellite sequences.

We manually reviewed the remaining insertions and identified other types of insertions (Fig. 4B, C): three processed pseudogenes from each of NA18943 and NA19240, five and six dispersed duplications, respectively, and 61 (1.1%) and 79 (1.2%) alternative sequences, respectively. For example, a processed pseudogene found in NA19240, sized 1115 bp (the breakpoint was at chr11:60,274,156), was precisely mapped to *RPLP0* on chromosome 12 with 99.9% in BLAT identity (Fig. 4D). This processed pseudogene represented a specific splice variant. Notably, we found 233 (4.2%) and 287 (4.4%) variants in NA18943 and NA19240 mapped to panTro6, although these variants did not exist in GRCh38 (“deletions” in GRCh38), suggesting that they had been caused by deletion events in the genomes composing the GRCh38 assembly [3]. We also found one NUMT insertion, sized 532 bp in NA19240.

After our manual review, 398 (7.2%) and 574 (8.8%) insertions remained to be assigned in NA18943 and NA19240, respectively (Fig. 4B, C). Of those insertions, 167 (3.0%) and 214 (3.3%) occurred in segmental duplications and self-chains in NA18943 and NA19240. The numbers of unallocated insertions were 231 (4.2%) and 360 (5.5%) (Fig. 4B).

The insertions mapped to alternative sequences tended to be large (median length = 4049 bp). In NA19240, 12 insertions (15%) mapped to the alternative sequences were not found in the standard SV set (Additional file 1: Table S9). For instance, an insertion sized 7332 bp, derived from an alternative sequence (chr6_GL000254v2_alt), was found in the LoMA SV set (the breakpoint was chr6:32,687,972). This breakpoint was located 15 kbp upstream from *HLA-DQB1* in the *HLA* region and showed a high sequence identity (99.8%) to chr6_GL000254v2_alt (Fig. 4E). However, the standard SV set by a previous study reported a translocation, not an insertion, in this region, suggesting an error call [25]. In NA18943, we found the longest insertion, sized 14,330 bp, at chr12:127,153,629 derived from an alternative sequence (chr12_KZ559112v1_alt). This insertion also showed a high sequence similarity (99.8% in identity) to the alternative sequence (Additional file 2: Fig. S6).

### Tandem repeats (TRs)

**Fig. 5:**
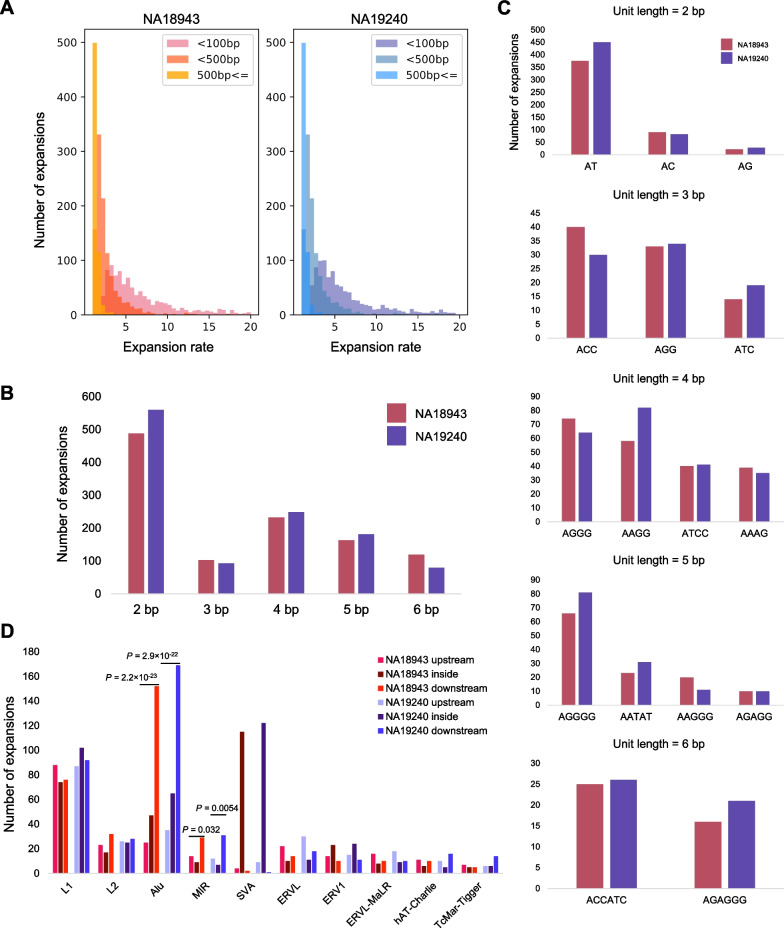
The variation of TR expansions.** A**. The expansion rate is shown up to 20. Left panel, NA18943; right panel, NA19240. Both panels are separated by the reference TR length (< 100 bp, < 500 bp, ≥ 500 bp). **B**. The number of variants of STRs (2–6 bp). **C**. The patterns of STR expansions are shown individually by unit length. Elements in both samples with a frequency of at least 10 are displayed. **D**. Sites of TR expansions surrounding TEs. Red, NA18943; blue, NA19240. Each class is separated into the upper stream (< 100 bp), lower stream (< 100 bp) and inside from the left. In SINE (*Alu* and *MIR*), TR expansions often occurred downstream of TE elements. SVAs contained relatively many expansions inside considering the fewer number than in the LINE family

Most TR insertions were found in the annotated TRs of GRCh38. The analysis of expansion rates showed that most TRs had low expansion rates (Fig. 5A), but high expansion rates were observed mainly in short TRs (< 100 bp) (Fig. 5A). The analysis of repeat elements showed that expansions of triplet repeats were rarer than 4- and 5-bp unit repeats in NA18943 and NA19240 (Fig. 5B). The expansions of (AT)*n* were major in 2-bp unit repeats, (ACC)*n* and (AGG)*n* in 3-bp, (AGGG)*n* and (AAGG)*n* in 4-bp, and (AGGGG)*n* in 5-bp (Fig. 5C). In each sample, 42% (NA18943) and 39% (NA19240) of TRs were found in genic regions, which was consistent with the size of the genic region in the human genome (Additional file 2: Fig. S7). Only 7 and 12 genes contained exonic TR expansions in NA18943 and NA19240, respectively (Additional file 1: Table S10). However, they were associated with various human traits and disease, such as the telomere length (Additional file 1: Table S10).

We also analyzed the association between TRs and TEs. We found that TR expansions around TEs were more likely to occur downstream of SINEs (*Alu* and *MIR*) than upstream (binomial test: *P* = 2.2 × 10^–23^ (*Alu*) and *P* = 0.032 (*MIR*) in NA18943; and *P* = 2.9 × 10^–22^ (*Alu*) and *P* = 0.0054 (*MIR*) in NA19240) (Fig. 5D). However, we did not see the same tendency in LINEs (L1 and L2) (Fig. 5D). In SVA elements, many TR expansions were observed inside compared with other families, such as LINEs, possibly because of the unique structure of SVA elements containing a VNTR region (Fig. 5D).

### Target site duplications (TSDs) in Alu elements

**Fig. 6:**
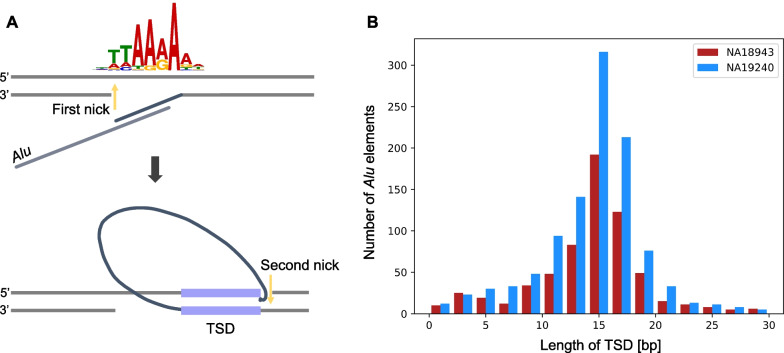
Analysis of TSDs among *Alu* insertions.** A**. The motif search using 1579 *Alu* elements by MEME Suite [35] confirmed the motif previously studied at the first nicking site. **B**. The length distribution of TSDs among *Alu* insertions, capped at 30 bp, binned per 2 bp. The distribution had a peak around 15 bp and was symmetrical in both samples

In NA18943 and NA19240, 1579 non-redundant *Alu* insertions were detected. The motif search of flanking sequences detected a strong motif at the first nicking site, TTAAAAA (*E*-value = 2.5 × 10^–315^) (Fig. 6A), as shown in previous studies [33, 34, 38]. On the other hand, no clear motif was identified around the second nicking site. We next analyzed the length distribution of TSDs flanking *Alu* elements, and single-peak distributions (median length of 15 bp) were obtained (Fig. 6B). The overall shape of the distributions was slightly skewed left, showing TSDs longer than 15 bp were fewer than TSDs shorter than 15 bp.

### Tandem duplications (TDs)

**Fig. 7:**
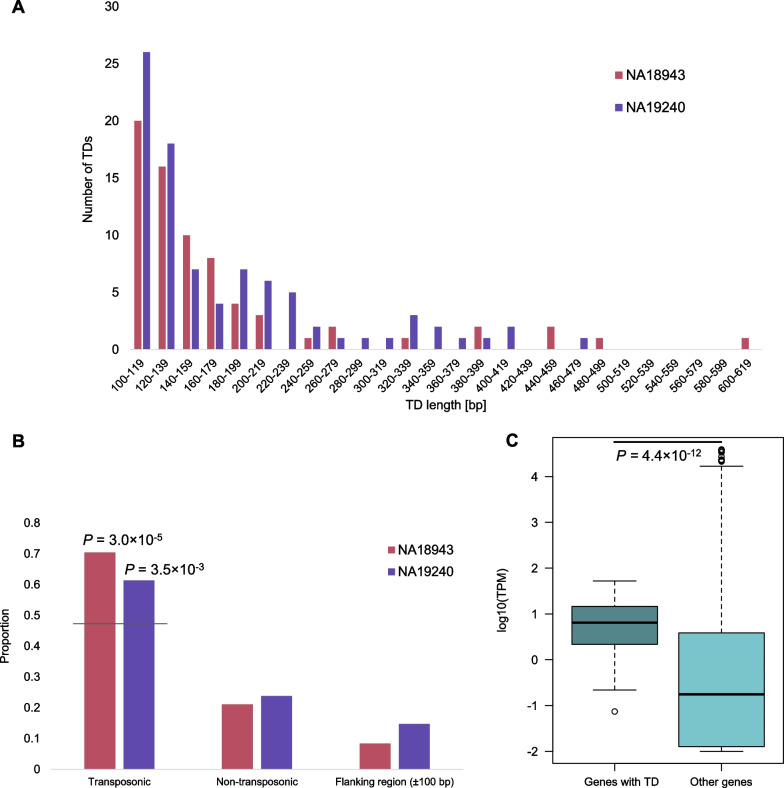
The characteristics of short TDs.** A**. The length distributions of TDs in NA18943 and NA19240. The number of TDs decreased as the length of TDs became longer.** B**. The enrichment of TDs in TEs. TDs were enriched in transposonic regions (79% in NA18943 and 76% in NA19240 when flanking regions are included). The gray line is the expected value of TDs in TE.** C**. The association of gene expression levels between genes with and without TDs. Genes containing TDs were expressed more than other genes. TPM: Transcripts Per Million

Lastly, we detected and analyzed TDs (*n* = 71 in NA18943 and *n* = 88 in NA19240). The length distribution showed that TDs were relatively short (the largest duplication was ~ 600 bp in length) and that short TDs were dominant (Fig. 7A). A manual review using UCSC Genome Browser [39] showed that 50 (NA18943) and 54 (NA19240) TDs were observed in TEs and significantly enriched in TEs in NA18943 and NA19240, respectively (binomial test: *P* = 3.0 × 10^–5^ in NA18943 and *P* = 3.5 × 10^–3^ in NA19240) (Fig. 7B). We analyzed the association of TDs and genes. TDs were observed in 30 (NA18943) and 34 (NA19240) genes, and together, 54 non-redundant TDs in genic regions were identified (Additional file 1: Table S11). The genes with TDs showed higher expression levels than other genes (Wilcoxon test: *P* = 4.4 × 10^–12^) (Fig. 7C), although TDs were not enriched in gene regions (Additional file 2: Fig. S8).

## Discussion

Assembling and polishing noisy long reads for local genomic regions can be a useful approach for the investigation of SVs underlying the human genome. This study showed that LoMA constructs accurate CSs from error-prone ONT reads and revealed fine structures of insertions. In this study, we evaluated the performance of LoMA using real and simulated data. A comparison with GRCh38 showed that LoMA reduced the error rate from 8.7% (raw ONT reads) to 0.76% (Fig. 3A). Sanger sequencing estimated the error rate of CSs by LoMA as 0.29% (Fig. 3B). In the simulation, the average error rate dropped below 0.1% with a coverage of 30×, which is an error rate comparable with short-read sequencing technologies (Fig. 3C). Additionally, most heterozygous SVs detected by LoMA were successfully validated by PCR (Additional file 2: Fig. S2), suggesting a good accuracy of haplotype representation. We also compared the accuracy with a similar tool, lamassemble, finding LoMA had a lower average error rate (Fig. 3A). Notably, the error patterns showed different patterns: the insertion rate was larger in LoMA in many cases (69%, 74 from 108 regions), although the deletion rate was larger in lamassemble in most cases (94%, 102 from 108 regions) (Additional file 1: Table S1). Thus, LoMA has the advantage of a lower deletion rate, although both tools may have systematic bias. Moreover, LoMA is superior in haplotype representation. Many de novo assemblers and lamassemble lack haplotype resolution, which results in representing a pseudo-haplotype and a decline in accuracy [12, 15]. Taken together, these results demonstrated that LoMA had sufficient accuracy to investigate genomic sequences at single-base resolution.

Since a large part of the human genome is identical within human populations [40], a targeted analysis of unclear regions should be enough for most human genome studies. In the current study, we focused on regions where clipped or collapsed reads were clustered (Fig. 2A), because such regions were likely to contain SVs and unstable genomic structures. The unclear regions were scattered throughout the entire genome, but the clusters were observed in the pericentromeric and *HLA* region, as expected (Fig. 2B).

We reconstructed the unclear regions using LoMA to understand their structures. Insertions are difficult to resolve because an inserted sequence complicates alignment and remains uncharacterized without an accuracy sequence. We found 5516 and 6542 insertions (≥ 100 bp) in NA18943 and NA19240, respectively (Fig. 4B) and restored them from single-platform data. This analysis identified some interesting examples that showed the effectiveness of our approach. First, a processed pseudogene of *RPLP0* (Fig. 4D) was accurately aligned to the original gene on chromosome 12 (99.9% in identity), although *RPLP0* has many reverse-transcribed copies scattered on other chromosomes. Among them, the identified insertion was most accurately aligned to the original gene (specific splice variant) rather than the other reverse-transcribed copies (the second most accurate alignment was 98.7% in identity), suggesting that the processed pseudogene was derived from the original *RPLP0* gene, not from other pseudogenes. This result indicated the importance of high-quality CSs for biological analyses. Second, we identified 15% of insertions of the alternative sequences in NA19240 were undetected in the standard SV set (Additional file 1: Table S9); these insertions were likely caused by analytical difficulties in a previous study [25]. Particularly, we corrected the SV structure (Fig. 4E) in the *HLA* region, which is known for its hypervariability in human populations [41]. In NA18943, an insertion (~ 14 kbp) derived from an alternative sequence was highly concordant (99.8% in identity) (Additional file 2: Fig. S6). Since such long insertions are difficult to restore by a mapping-based approach, this result indicates the efficiency of our assembly approach.

Our analysis showed that TR expansions were highly variable in length (Fig. 5A). Large TR expansions found in our analysis ranged thousands of base pairs. Although the landscape of TR expansions has been studied using short and long reads [42–44], to our knowledge, no studies have constructed long repeats and assessed the expansion rate throughout genomes. Many studies have identified pathogenic long repeat expansions [2, 44], and several methods were developed to detect repeat expansions. However, such laboratory techniques are not suitable for multiple loci [44]. Thus, genome-wide analysis using LoMA will help explore TR expansions more widely and simply. Further, our analysis suggested that TR expansions existed even in healthy individuals, and these polymorphisms may affect disease susceptibility. Indeed, our results showed healthy individuals had expansions in exons consisting of TRs, and most of them were associated with various human traits and disease susceptibility (Additional file 1: Table S10) [45, 46]. Further studies of human populations using long reads may identify disease-related TR expansions. We also observed TR expansions inside TEs, especially in SVAs (Fig. 5D), suggesting the existence of repetitive polymorphisms in repetitive sequences. A recent study reported SVs in SVAs associated with neurological disorders [47]. Our results are consistent with this previous study and indicated the importance of investigating TR expansions in TEs. Nested repeats are hard to identify without long reads, and an assembly approach is necessary to interpret them. Therefore, investigations of human genetic variations using our method may lead to findings of novel susceptible genes of complex diseases. We also showed that TR expansions are prone to exist downstream of SINEs (*Alu* and *MIR*) (Fig. 5D). This relationship may reflect the reverse transcription mechanism in which an RNA element introduces a poly(A) sequence into the inserted site and provide a source of genomic instability around the site that gives rise to TR expansions.

*Alu* elements are non-autonomous retrotransposons, and their insertion is initiated by target site-primed reverse transcription dependent on L1 endonuclease and reverse transcriptase, although there is no consensus on how the synthesized end is integrated to the target sequence on the opposite DNA strand [34, 48]. We showed that the first nick induced by the initiation of the target-site primed reverse transcription of an *Alu* element has a strong motif, which is consistent with sites that L1 ORF2p endonuclease recognizes [49]; however, our analysis also suggested this recognition can take place at other sites that contain G-rich sequences (Fig. 6A). The length of a TSD is dependent on the second nicking site in the sense strand [34]. A past study suggested that TSD lengths are within 15–16 bp, though the investigated size of an *Alu* element was relatively small [50]. Our results corroborated that finding and further suggested that the distribution was skewed left, with an average length of 15 bp (Fig. 6B).

Lastly, we showed the characteristics of TDs. This class of insertions is understudied due to a lack of genome-wide sequence analysis at single-base resolution. We found that genes containing a TD were highly expressed compared to other genes (Fig. 7C), suggesting that TDs are induced by transcription stress. Furthermore, TDs were enriched in transposable elements (Fig. 7B). It is known that inversions are induced in L1 retrotransposition [51]. TE integration may also be related to the birth of novel TDs by some mechanism through the reverse transcription process. In the present study, we did not analyze indels shorter than 100 bp in length. However, the length distribution of TDs suggested that a number of TDs lie in short ranges (Fig. 7A) [29]. The numerical dominance of short TDs may contribute to genetic variations in the human population.

We revealed the true structures of insertions with high accuracy and inferred mechanisms for the insertions. However, our study has several limitations to be addressed in the future. First, LoMA classified haplotypes based on SVs, but SNVs and short indels were not taken into account. This is because current long reads have high sequencing error rates and haplotype classification based on SNVs may cause computational errors. As sequencing technologies and basecalling accuracies improve, SNV-based haplotype reconstruction should be possible in the near future. Second, SV callings in low-coverage regions had low precision (Fig. 2C) due to high error rates in the current platforms. Improvements in sequencing error rates will enable reliable data from low-coverage regions. Third, in the genome-wide survey of unclear regions, we could not obtain satisfactory results in centromeric satellites and other complex regions, because the lengths of the reads were not sufficient to completely resolve these complex regions (Additional file 1: Table S3) [19]. Longer reads should help to analyze these regions.

## Conclusions

Localized assembly using long reads is a promising approach to explore genetic variations in human populations. We demonstrated the effectiveness of our approach by revealing the true structures of insertions and repetitive regions at single-base resolution. Applications of this approach to human disease studies will enable us to find novel pathogenic variants of Mendelian and complex disorders.

## Supplementary Information

**Additional file 1:** Supplementary tables.

**Additional file 2:** Supplementary materials and figures.

## Data Availability

The sequencing data have been deposited in DDBJ under the accession number DRR450710 [52]. The source code can be downloaded from the author’s GitHub page: https://github.com/kolikem/loma [53].

## Abbreviations

ONT: Oxford nanopore technologies
PacBio: Pacific biosciences
SV: Structural variant
TR: Tandem repeat
TE: Transposable element
T2T: Telomere-to-telomere
CS: Consensus sequence
WGS: Whole-genome sequencing
TD: Tandem duplication
NUMT: Nuclear mitochondrial DNA sequence
TRF: Tandem repeats finder
UTR: Untranslated region
STR: Short tandem repeat
TSD: Target site duplication
SD: Standard deviation
