# Supplementary material for: Localized assembly for long reads enables genome-wide analysis of repetitive regions at single-base resolution in human genomes
Source: Hum Genomics. 2023 Mar 9;17:21. doi: 10.1186/s40246-023-00467-7 (PMC9996862; doi:10.1186/s40246-023-00467-7)
Supplement: Supplementary file 2 — Additional file 2: Supplementary materials and figures. [file 40246_2023_467_MOESM2_ESM.docx]

**Supplementary Materials**

**Determination of thresholds for identifying mismatch clusters**

LoMA detects heterozygous SVs in a target region and outputs haplotype-resolved sequences. To detect heterozygous SVs, all input reads are aligned to the first CS using minimap2, and heterozygous SVs are detected based on the number of discordant reads. The number of reads containing a heterozygous SV is expected to follow a binomial distribution with *n* (coverage) and *p* = 0.5. If the coverage is sufficiently high, the binomial distribution approximates a normal distribution with mean *n*/2 and variance *n*/4. Using the mean and standard deviation (σ) of the normal distribution, we calculated the expected range of the number of discordant reads. We additionally used a threshold for the minimum number of discordant reads. In this analysis (~80x coverage), the range of the number of discordant reads and the minimum number of discordant reads were set to the mean ± 3σ and 8, respectively, respectively. These thresholds can be changed according to the amount and characteristics of the user’s data.

**Counting STR types**

In principle, it is difficult to distinguish in which strand a TR expansion event occurred by observing DNA sequences. Additionally, it is also difficult to analyze STRs tandemly repeated many times after discriminating sorted sequences that keep their order (e.g., CGA, ACG and GAC). Therefore, we treated and counted a group of STRs that were the same in lexicographical order without discriminating strands as the same group.

**Genome-wide analysis of SVs using LoMA**

The success rate of the reconstruction of unclear regions was 88.1% and 92.2% in NA18943 and NA19240, respectively, excluding the failed regions derived from centromeric regions. In NA18943, 3,065 regions were classified as heterozygous, and 10,757 were homozygous regions. In NA19240, 7,542 and 7,678 regions were heterozygous and homozygous, respectively. The difference in the ratio of heterozygous and homozygous regions reflected the high genetic diversity in the African population. We then assessed the success rate of LoMA in regions in which clipped sequences accumulated, because sequence information is not accessible in regions full of clipped reads and thus clipped sequences are ignored in typical analyses. In NA18943 and NA19240, 82.5% and 71.8%, respectively, of the clipped-sequence-accumulating regions were correctly assembled excluding centromeres.

**Fig. S1**: Quality control in data preprocessing. Read pairs with a dangling read and those with short alignment lengths are excluded. Read pairs that pass these filters are used to construct a read layout.

**Fig. S2:** Examples of PCR validation. Six of eight heterozygous variants validated by PCR amplification are shown in the picture of an electropherogram. The numbers are the variant IDs in Table S4. White arrows indicate the amplified products in expected sizes. The asterisks are non-specific bands.

**Fig. S3**: The error rate of assembled sequences in the simulated data (n = 100 for each coverage). The mean coverage and error rate were correlated. The average error rates were 2.6% (10x), 0.29% (20x), 0.076% (30x), 0.041% (40x), and 0.034% (50x) for each coverage.

**Fig. S4**: The computation performance of LoMA. **A** and **B** show the change in average computation time for different mean coverages and target sizes, respectively (n = 100). **C** and **D** show the change in average peak resident set size (RSS) for different mean coverages and target sizes, respectively (n = 100). The data in **A** and **C** are shown in increments of 10 from 10x to 50x. The data in **B** and **D** are shown in increments of 20 kbp from 20 kbp to 100 kbp. Each dot is the average of one hundred regions.

**Fig. S5**: Boxplots of the computation performance of LoMA. **A** and **B** show the boxplots of average computation times for different mean coverages and target sizes, respectively (n = 100). **C** and **D** show the boxplots of average peak RSS for different mean coverages and target sizes, respectively (n = 100). The data in **A** and **C** are shown in increments of 10 from 10x to 50x. The data in **B** and **D** are shown in increments of 20 kbp from 20 kbp to 100 kbp.


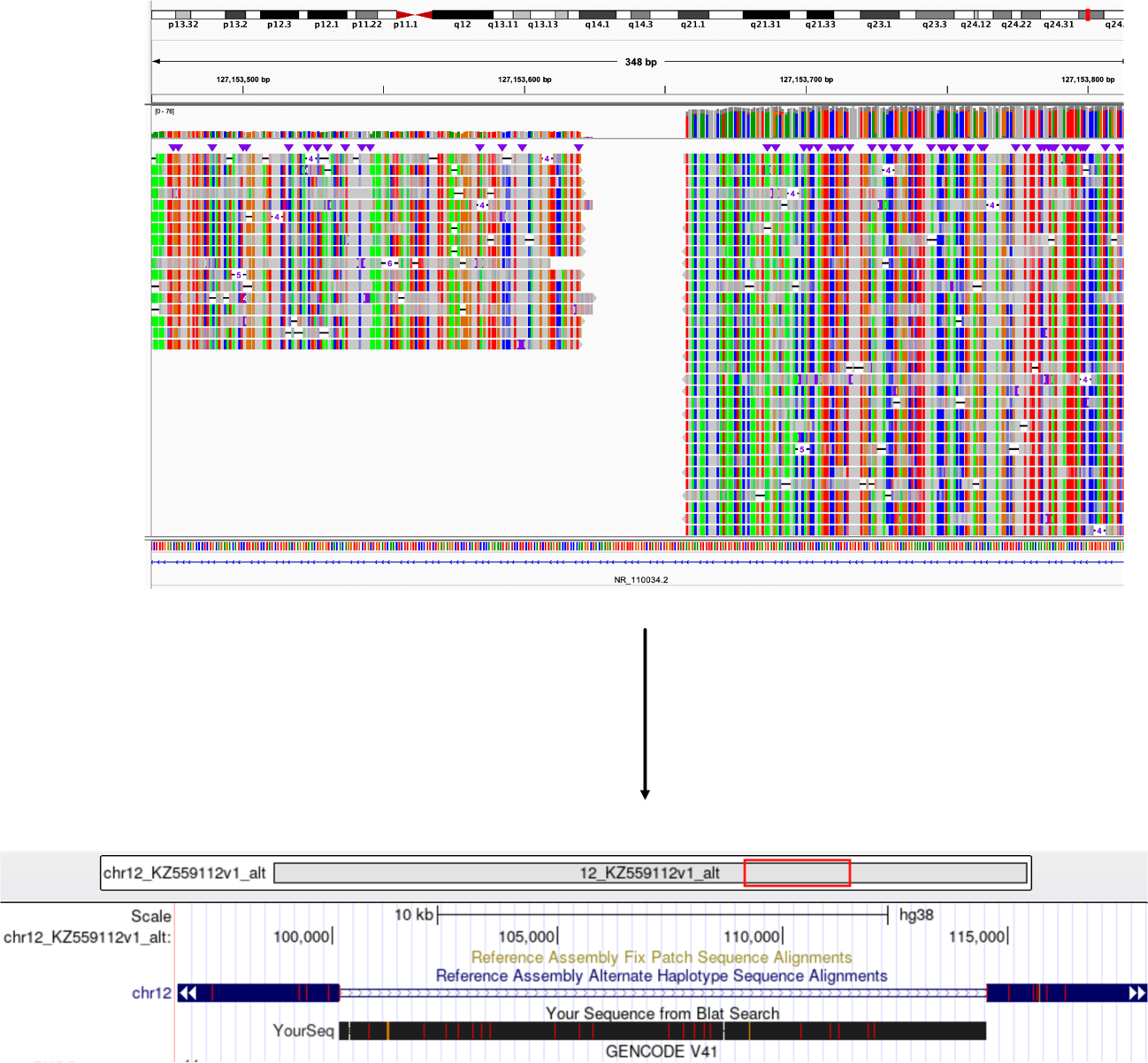


**Fig. S6:** The longest alternative sequence insertion. A long insertion (~14 kbp) that accurately mapped (99.8% in identity) to an alternative sequence on chr12 was detected in NA18943. It showed the clip-accumulated pattern, as visualized by IGV [1]. After the restoration of this region, the sequence was clearly mapped to the alternative sequence on Genome Browser [2].


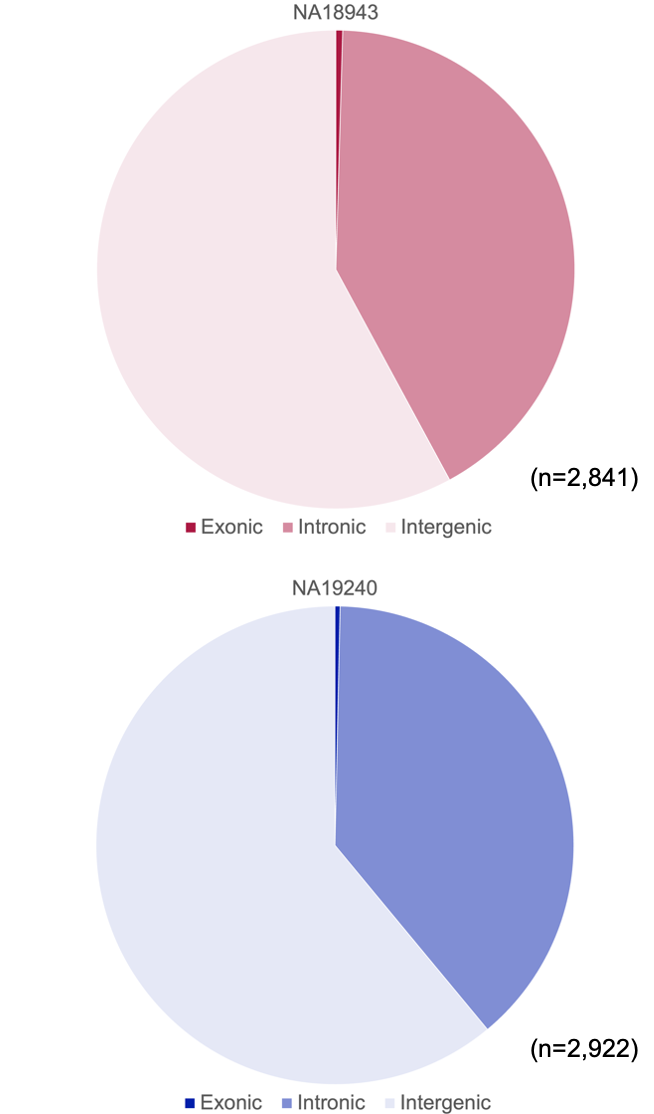


**Fig. S7:** Genic and intergenic TR expansions. In NA18943, genic TDs accounted for 42%, and in NA19240 they were 39%, which is consistent with the size of the genic region in the human genome. Only 7 and 12 genes contained exonic TR expansions in NA18943 and NA19240, respectively.


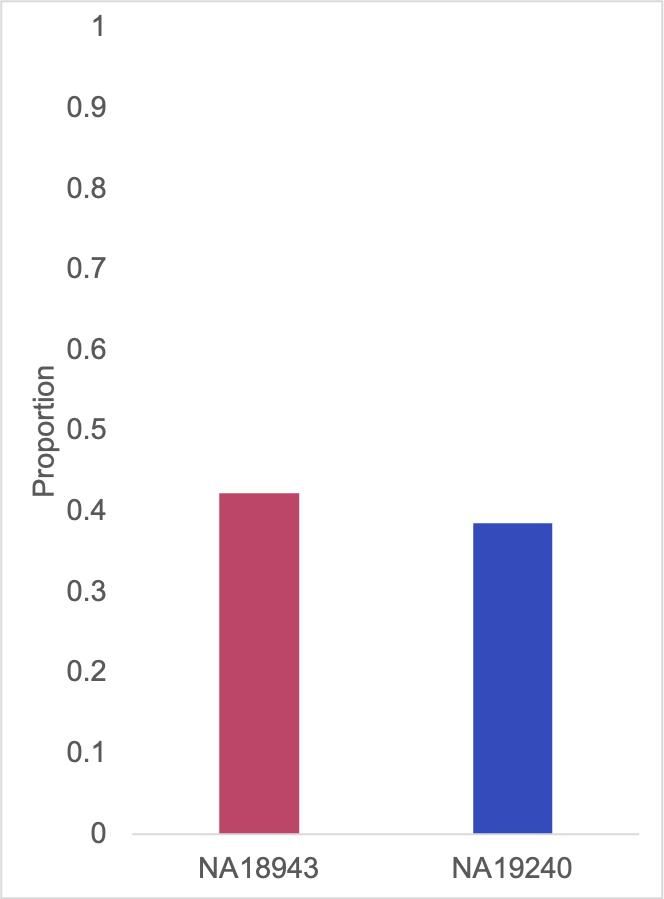


**Fig. S8:** Genic TDs. In NA18943, 30 TDs (42%) occurred in the genic region (all in non-CDS regions); in NA19240, 34 TDs (39%) occurred in the genic region (all in non-CDS regions). These observations are consistent with the size of the genic region in the human genome. TDs were not enriched in genes.

**References**

﻿1. Thorvaldsdóttir H, Robinson JT, Mesirov JP. Integrative Genomics Viewer (IGV): High-performance genomics data visualization and exploration. Brief Bioinform. 2013;14:178–92. https://doi.org/10.1093/bib/bbs017.

2. Genome Browser. <https://genome.ucsc.edu/index.html>. Accessed 17 October 2022.
